# A functional definition to distinguish ponds from lakes and wetlands

**DOI:** 10.1038/s41598-022-14569-0

**Published:** 2022-06-21

**Authors:** David C. Richardson, Meredith A. Holgerson, Matthew J. Farragher, Kathryn K. Hoffman, Katelyn B. S. King, María B. Alfonso, Mikkel R. Andersen, Kendra Spence Cheruveil, Kristen A. Coleman, Mary Jade Farruggia, Rocio Luz Fernandez, Kelly L. Hondula, Gregorio A. López Moreira Mazacotte, Katherine Paul, Benjamin L. Peierls, Joseph S. Rabaey, Steven Sadro, María Laura Sánchez, Robyn L. Smyth, Jon N. Sweetman

**Affiliations:** 1 Biology Department, State University of New York at New Paltz, New Paltz, NY USA; 2 Department of Ecology and Evolutionary Biology, Cornell University, Ithaca, NY USA; 3 School of Biology and Ecology, Climate Change Institute, University of Maine, Orono, ME USA; 4 Departments of Biology and Environmental Studies, St. Olaf College, Northfield, MN USA; 5 Department of Fisheries and Wildlife, Michigan State University, East Lansing, MI USA; 6 Instituto Argentino de Oceanografía (IADO), Universidad Nacional del Sur (UNS)-CONICET, Florida 8000, Complejo CCT CONICET Bahía Blanca, Edificio E1, B8000BFW Bahía Blanca, Argentina; 7 Centre for Freshwater and Environmental Studies, Dundalk Institute of Technology, Dundalk, Ireland; 8 Department of Fisheries and Wildlife and the Lyman Briggs College, Michigan State University, East Lansing, MI USA; 9 Department of Geography, York University, Toronto, ON Canada; 10 Department of Environmental Science and Policy, University of California, Davis, Davis, CA USA; 11 National Scientific and Technical Research Council (CONICET), Cordoba, Argentina; 12 Battelle, National Ecological Observatory Network (NEON), Boulder, CO USA; 13 Department of Ecohydrology and Biogeochemistry, Leibniz-Institute of Freshwater Ecology and Inland Fisheries (IGB), Müggelseedamm 310, 12587 Berlin, Germany; 14 Lakes Environmental Association, Bridgton, ME USA; 15 Department of Ecology, Evolution, and Behavior, University of Minnesota-Twin Cities, St. Paul, MN USA; 16 CONICET-Universidad de Buenos Aires, Buenos Aires, Argentina; 17 Environmental and Urban Studies, Bard College, Annandale-on-Hudson, NY USA; 18 Department of Ecosystem Science and Management, Penn State University, University College, PA USA

**Keywords:** Freshwater ecology, Ecology, Environmental sciences, Limnology

## Abstract

Ponds are often identified by their small size and shallow depths, but the lack of a universal evidence-based definition hampers science and weakens legal protection. Here, we compile existing pond definitions, compare ecosystem metrics (e.g., metabolism, nutrient concentrations, and gas fluxes) among ponds, wetlands, and lakes, and propose an evidence-based pond definition. Compiled definitions often mentioned surface area and depth, but were largely qualitative and variable. Government legislation rarely defined ponds, despite commonly using the term. Ponds, as defined in published studies, varied in origin and hydroperiod and were often distinct from lakes and wetlands in water chemistry. We also compared how ecosystem metrics related to three variables often seen in waterbody definitions: waterbody size, maximum depth, and emergent vegetation cover. Most ecosystem metrics (e.g., water chemistry, gas fluxes, and metabolism) exhibited nonlinear relationships with these variables, with average threshold changes at 3.7 ± 1.8 ha (median: 1.5 ha) in surface area, 5.8 ± 2.5 m (median: 5.2 m) in depth, and 13.4 ± 6.3% (median: 8.2%) emergent vegetation cover. We use this evidence and prior definitions to define ponds as waterbodies that are small (< 5 ha), shallow (< 5 m), with < 30% emergent vegetation and we highlight areas for further study near these boundaries. This definition will inform the science, policy, and management of globally abundant and ecologically significant pond ecosystems.

## Introduction

**Figure 1:**
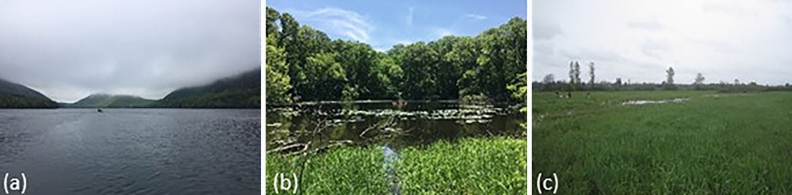
We call lentic waterbodies by a variety of names in the English language including ponds, lakes, wetlands, reservoirs, oxbows, prairie potholes, vernal pools, lagoons, dams, puddles, and shallow lakes. These names may or may not correspond to ecological and systematic differences. Generally, laypeople and experts, as individuals, will quickly differentiate among broad categories of ponds, lakes, and wetlands; however, individuals may respond in different ways depending on their background and experiences. We present three different images of waterbodies that could each be categorized as lake, pond, or wetland using objective (e.g., morphology or vegetative cover) or more subjective criteria keeping cognizant of the complexity within and potential overlap among waterbody types.

Lentic (still) waterbodies have long been placed into categories to improve our understanding of aquatic ecosystems, aid science communication, and facilitate management decisions^1,2^. For instance, lentic ecosystems have been sorted into discrete categories by size or depth^3,4^, trophic status^5^, and mixing regime^6,7^. Often, lentic waterbodies are categorized by different ecosystem types, such as lakes, ponds, and wetlands (Fig. 1). Categorizing waterbodies using physical and biological characteristics facilitates generalizations and decision making, but categories may not always align with ecological inferences^2^.

Categorizing small waterbodies is particularly challenging. The majority of the world’s lentic waterbodies are small: over 95% are less than 10 ha (0.1 km^2^)^3,8^. Across the history of limnology, small and shallow waterbodies are widely referred to as ponds^8–11^, yet pond definitions differ across the globe and are not based on scientific evidence. The lack of a universal, scientifically-based pond definition that differentiates ponds from other lentic waterbodies hampers science, policy, and management, and creates confusion. For example, the number of lakes at regional and global scales is contested and depends on the lower bounds of what is considered a “lake”^3^. In the United States (US), Wisconsin and Minnesota debated which state had the most lakes based on Wisconsin including smaller waterbodies < 0.1 ha as lakes, whereas Minnesota considered these small waterbodies as wetlands^12–14^. These definitions affect which waterbodies are included in monitoring programs, and how ecosystem properties are regionally or globally upscaled. For instance, ponds are often grouped with lakes when upscaling greenhouse gas emissions^4^, but there is concern over double counting ponds as wetlands and thus overestimating aquatic emissions^15^. These examples emphasize the importance of waterbody categorization for science, management, and legal protection.

Distinguishing ponds from lakes and wetlands is common among the public, scientists, and managers. Yet, while scientists speculate that ponds may be fundamentally different in ecosystem structure and function compared to lakes and wetlands^16^, these data have not been collected and analyzed with the explicit purpose of defining boundaries between aquatic ecosystem types. Therefore, our study had four objectives: (1) compile current pond definitions from scientists and policy makers, (2) determine if ponds, lakes, and wetlands, as defined by researchers, differ in ecosystem structure, (3) use ecosystem structure and function metrics to identify if there are boundaries between ponds and lakes or wetlands, and (4) develop a scientifically based pond definition based on ecosystem function and prior definitions. To address our objectives, we compiled existing pond definitions from scientific literature and evaluated legislative definitions of ponds, wetlands, and lakes. We also assembled a large dataset of pond characteristics and ecosystem function from a global literature survey and compared ecosystem structural and functional metrics among ponds, wetlands, and lakes. Finally, we propose an evidence-based pond definition.

## Results and discussion

### Current scientific definitions of ponds

**Figure 2:**
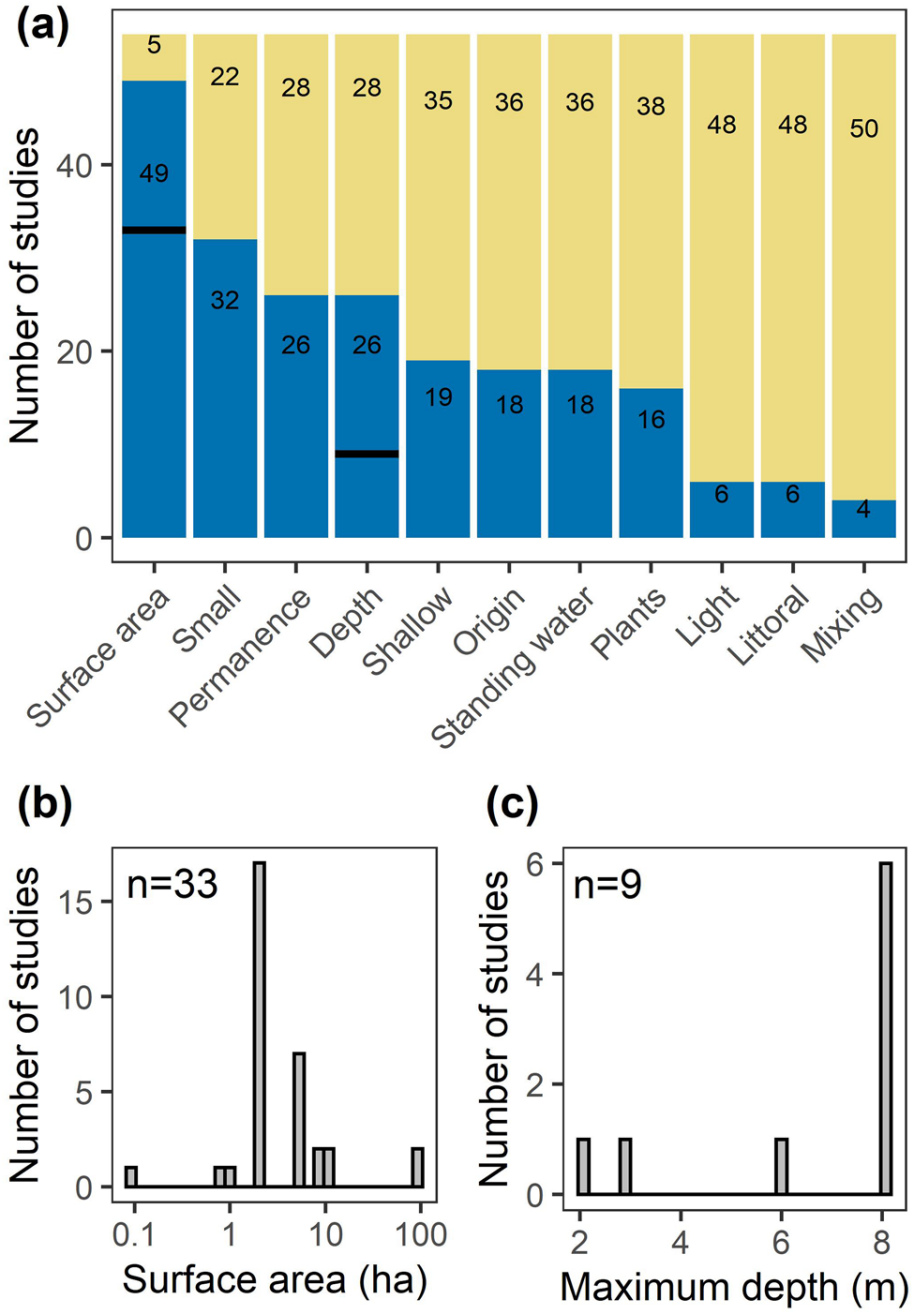
Summary of “pond” definitions from scientific literature including (**a**) presence of various morphological, biological, and physical characteristics in the definition as blue bars (n = 54 definitions total). Bold black lines indicate the number of definitions with surface area and depth values. Histograms of the upper limits from “pond” definitions for (**b**) surface area and (**c**) maximum depth.

We compiled existing scientific definitions of ponds by conducting a backwards and forwards search of papers referenced in or subsequently referencing three seminal pond papers^8,17,18^ (see “Methods”). We ultimately compiled 54 pond definitions from scientific literature (data available^19^). The variables most often included in definitions were surface area (91% of definitions), depth (48%), permanence (48%), origin (i.e., natural or human-made; 33%), and standing water (33%; Fig. 2a). When surface area or depth were included in definitions, they were often mentioned qualitatively (e.g., “small” and “shallow”). Of the 61% of definitions that included a maximum pond surface area, the range was 0.1 to 100 ha, the median was 2 ha, and all but two definitions were ≤ 10 ha (Fig. 2b). For depth, only 17% of studies provided a maximum depth cutoff, which ranged 2 to 8 m (Fig. 2c). Of the 26 definitions mentioning permanence, 22 stated that ponds could be temporary or permanent and only three indicated that ponds are exclusively permanent waterbodies. Of the 18 definitions mentioning origin, 17 mentioned that ponds could be natural or human-made with the remaining study indicating ponds can have diverse origins.

Other important factors included in definitions related to morphometry. For example, 30% of definitions mentioned the potential for plants to colonize the entire basin, which relates to high light penetration (mentioned in 11% of definitions) and/or shallow depths. For example, Wetzel^11^ defines ponds as having enough light penetration that macrophyte photosynthesis can occur over the entire waterbody. As such, these conditions may be comparable to the littoral region of lakes (11% of definitions). Lastly, 7% of pond definitions mentioned mixing versus stratification, whereby ponds mix more than lakes^20^ yet less than shallow lakes due to a smaller fetch^16^.

To assess if there was agreement in pond definitions among papers, we examined the number of times each definition was cited. Across the 54 definitions, there were 89 citations of 48 unique papers. Ultimately, most papers (75%) were only cited only once, indicating no consensus in pond definition. The most cited paper was Biggs et al.^21^, which accounted for 15% of citations. The next two most cited papers were Oertli et al.^17^ and Sondergaard et al.^18^, which were seminal papers included in our backwards-forwards search, and each comprised 8% of citations.

### International definitions

At an international level, there is no consensus on how to discriminate among ponds, lakes, and wetlands. In North America, wetlands are generally considered to be shallow: < 2 m in Canada^22^ and < 2.5 m in the US^23^, which differentiates them from lakes. Some nations, such as Australia, South Korea, and Uganda, explicitly include ponds and lakes in federal wetland definitions^24^ (see also^22^). The inclusion of ponds and some lakes within wetland definitions often stems from the Ramsar Convention, an international body interested in global wetland conservation that has been signed by 172 countries representing 6 continents as of 2021^25^. The Ramsar Convention defined wetlands as “areas of marsh, fen, peatland, or water” across marine, brackish, and freshwater with varying degrees of permanence and natural or artificial states with a maximum depth of 6 m^26^, which overlaps depths found in many definitions of ponds and shallow lakes. In other countries, ponds are included in lake definitions under federal conservation laws. For example, in the Danish “nature protection” law §3, lakes are defined as waterbodies with a surface area of > 100 m^2^. As 98% of Danish ‘lakes’ are smaller than 1 ha^27^, this law protects many small waterbodies that may be considered ponds elsewhere. Still other agencies have only qualitative pond definitions: the European Commission simply defines ponds as “relatively shallow” and may also be called “pool, tarn, mere, or small lake,” a definition also used by the International Union for Conservation of Nature^28,29^. These examples underscore that waterbody definitions vary globally, are generally qualitative, and are rarely based on scientific evidence relating to ecosystem structure or function. The definitions possibly derive from different management, protection, and monitoring strategies; for instance, the European Union’s Water Framework Directive excludes waterbodies < 50 ha (0.5 km^2^) in size from monitoring^30^.

### Current U.S. Federal and State definitions

In the US, waterbody definitions vary among federal agencies, with implications for both legal protection and monitoring. The US Environmental Protection Agency (EPA) and the US Army Corps of Engineers (ACE) define wetlands based on saturated soils and hydrophytic vegetation, which has the potential to include ponds within the category of wetlands. Conversely, the US Fish and Wildlife Service (USFWS) distinguishes among wetlands and lakes based on surface area, depth, and emergent vegetation^31^. Lakes are ≥ 8 ha or if smaller, they must be ≥ 2.5 m in maximum depth. In contrast, wetlands are are dominated by > 30% emergent plant cover; if there is less, the site may still be a wetland if < 2.5 m deep and < 8 ha in size. Therefore, ponds are often considered by USFWS to be wetlands, but this is not always the case: ponds have been used as an example of a waterbody that can be classified as lake, wetland, or both^32^. The lack of an explicit, unified, and scientifically based pond definitions across three federal agencies (EPA, ACE, USFWS) is confusing and contributes to ponds being underrepresented in US aquatic waterbody monitoring relative to their numerical dominance on the landscape^3,8^. For example, US EPA monitoring programs include ponds in both the National Wetland Condition Assessment and the National Lake Assessment; however, “ponds” represent a small number of waterbodies in each of these surveys (< 12% classified qualitatively as “pond” in 2011 wetland survey; 13% of waterbodies were < 5 ha in 2012 lake survey).

**Figure 3:**
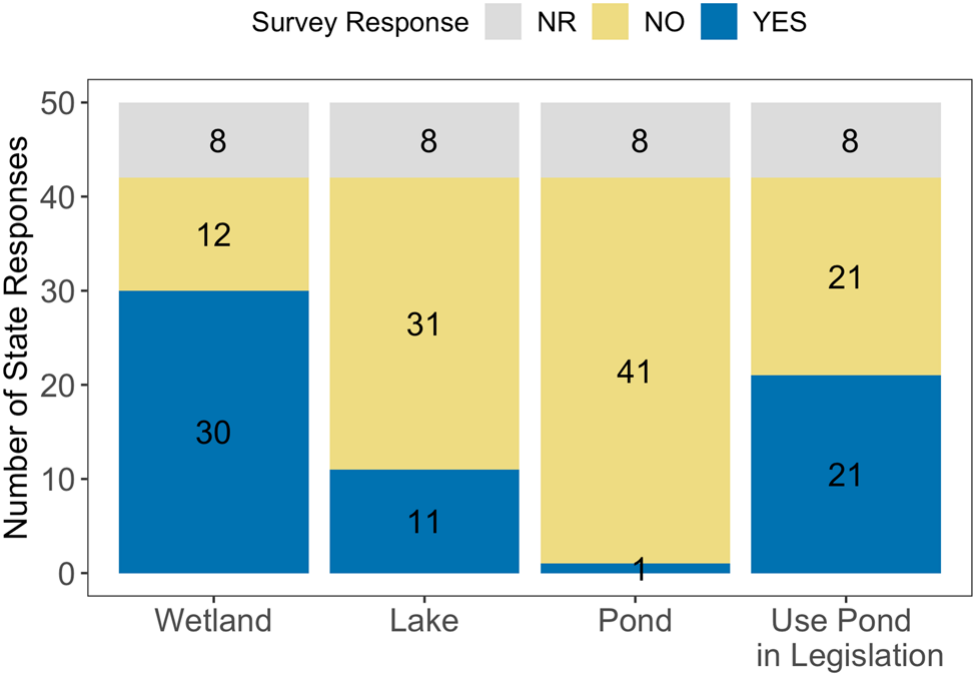
US state responses to surveys indicating if the state has a definition of wetland, lake, or pond and if the state used the term “pond” in their legislation. NR = no response.

Reflecting political and geographic variability at the national scale, most US states have their own waterbody protections^33^. We surveyed US state agencies to examine state definitions of ponds, lakes, and wetlands (see “Methods”). Our survey responses included 42 of 50 (84%) states (Fig. 3). Only one state (Michigan) explicitly defined ponds, 11 states defined lakes (26%), and 30 states defined wetlands (71%). While only one state defined ponds, half of the surveyed states used the term “pond” in their legislation. Specifically, ponds were referenced as state waters (e.g., Vermont) or were included in state definitions for lakes (e.g., Kansas) or wetlands (e.g., Rhode Island). It is unclear how these definitions impact monitoring and protection or why the distinctions were originally made. For instance, many states monitor lakes based on minimum size thresholds, which vary widely from < 1 ha in Arizona and Alaska, 2–4 ha in many northeastern states, and up to 8 ha in Washington and Nebraska. The variety of definitions and monitoring size cutoffs do not appear to be scientifically based, but may stem from arbitrary decisions, historic references, mapping capabilities from decades ago, and resource limitations for monitoring; the same rationale for definitions likely apply to local, regional, and international organizations around the globe.

### Comparing lake, pond, and wetlands characteristics from literature

We compared biological, physical, and chemical characteristics of waterbodies that scientists called lakes, ponds, or wetlands in published studies. To obtain data for the pond characteristics, we used the same literature search summarized above for pond definitions (also, see “Methods”). From the 519 papers that we examined, we extracted data on sites the authors called “ponds” and other variants (e.g., ‘small ponds’, ‘fish ponds’, but NOT ‘lakes’). We filtered waterbodies that were ≤ 20 ha surface area and ≤ 9 m depth (global distribution; n = 1327) to include waterbodies slightly greater than the maximum depth and maximum surface area used to define ponds in prior studies^34,35^. To compare ponds to lakes and wetlands, we used existing lake (US and Europe; n = 55,173) and wetland (US; n = 400) databases; waterbodies were classified as lake or wetland by the scientists or managers who published the database. Wetlands were classified as < 1 m in depth with no defined surface area and lakes were all > 0.02 ha with no defined depth (see “Methods'' for details).

From the waterbodies that scientists called “ponds,” hydroperiod and origin varied over a large range of characteristics. Of the 608 ponds with hydroperiod data, permanent ponds accounted for 74% (n = 450) and temporary ponds for 26% (n = 158). Out of 648 ponds with known origins, 65% (n = 418) were constructed or manipulated and 35% (n = 230) were natural. Therefore, scientists consider ponds to be inclusive of both permanent and temporary hydroperiod and have natural or human-made origins.

**Figure 4:**
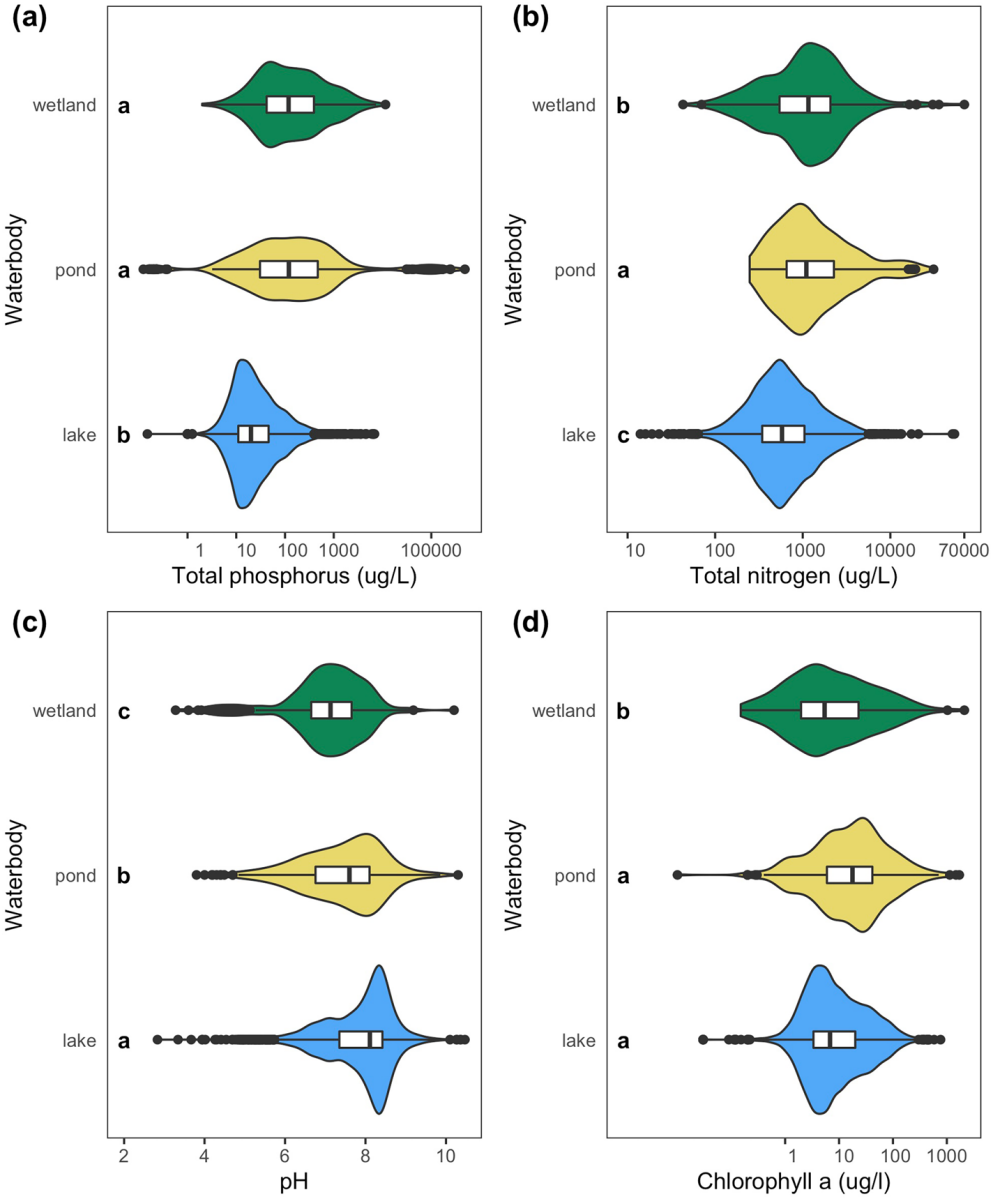
Comparison of various chemical and biological parameters across wetlands, ponds, and lakes, with waterbody category based on the term used by publishing scientists and managers (Table S2). Violin plots indicate distributions of waterbody characteristics, the white box indicates 25th to 75th percentile with median in the middle, whiskers indicate 1.5 × interquartile range, and outliers are black closed circles. Letters inside the plot indicate significant differences in means (LSD, alpha = 0.05). Note all x-axes have logarithmic scales.

When examining water chemistry, nutrients, and biotic data across different waterbody types, as defined by publishing scientists and managers, we found that ponds were distinct from lakes and wetlands in two metrics (TN, pH), similar to wetlands in one metric (TP), and similar to lakes in one metric (chl *a*; Fig. 4; Tables S1, S2). For example, ponds had distinctly high TN concentrations, which were greater than either lakes or wetlands (Fig. 4b; Table S2). Ponds and wetlands had similarly high TP concentrations, which were significantly greater than lakes; ponds were also most variable in TP (Fig. 4a; Table S2). Lastly, ponds chlorophyll (chl) *a* concentrations were similar to lakes, with wetlands being most variable but lower, on average (Fig. 4d; Table S2).

### Does ecosystem structure and function distinguish ponds from lakes and wetlands?

We evaluated the relationship between key metrics of ecosystem structure or function with three quantitative variables that often showed up in pond, lake, or wetland definitions: surface area, maximum depth (hereafter depth), and emergent vegetation cover. Our metrics of ecosystem structure or function include nutrients (total phosphorus (TP), total nitrogen (TN)), water chemistry (pH), primary producer biomass (chl *a*), metabolism (gross primary production—GPP, respiration—R, net ecosystem production—NEP), and heat and gas distributions and movement (diel temperature ranges—DTR, methane fluxes, gas transfer velocities). The data was collated from global surveys of literature and federal or international databases (see “Methods”) with ultimately ten comparisons for surface area, six comparisons for depth, and four comparisons for emergent vegetation cover with a range of sample sizes for each comparison (n = 67 to 7931, see Tables S3, S5, S7). We assessed each relationship for four different patterns in increasing order of complexity: null, linear, segmented (nonlinear), and logistic (nonlinear) patterns and selected the best fit and most parsimonious relationship.

**Figure 5:**
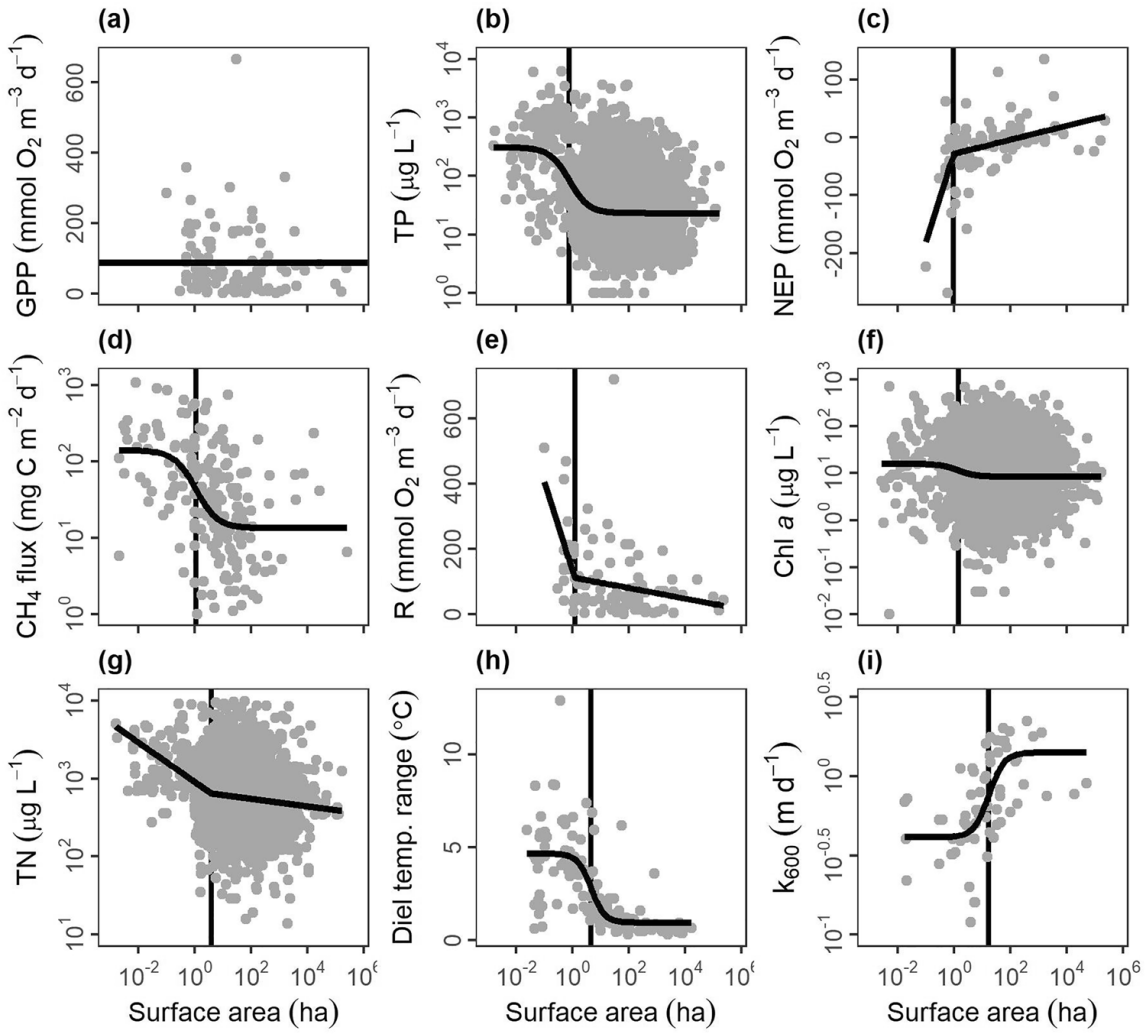
Relationships between lentic waterbody size (excluding wetlands) and ecosystem structure and function metrics: (**a**) gross primary production (GPP), (**b**) total phosphorus concentrations (TP), (**c**) net ecosystem production (NEP), (**d**) methane fluxes (CH_4_ flux), (**e**) respiration (R), (**f**) chlorophyll a concentrations (Chl a), (**g**) total nitrogen concentrations (TN), (**h**) diel temperature ranges (DTR), and (**i**) gas transfer piston velocity (k_600_). Optimal model fits from null, linear, segmented, and logistic curves in bold foreground lines. For nonlinear segmented and logistic models (**b**–**i**), plots are ordered by boundaries between ponds and lakes, as defined by model breakpoints or inflection points (vertical background lines).

**Figure 6:**
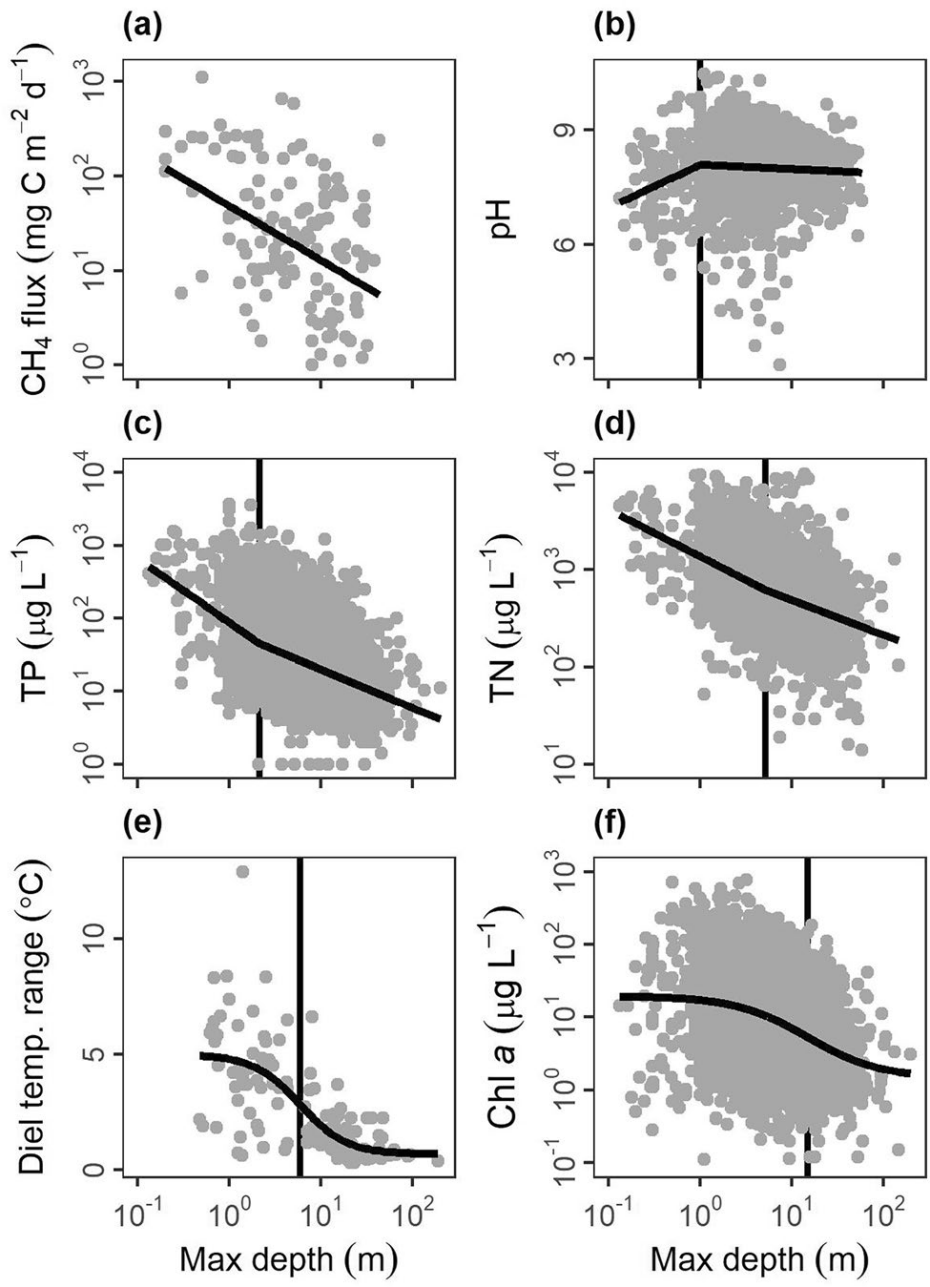
Relationships between lentic waterbody maximum depth (Max depth) and various ecosystem structure and function metrics: (**a**) methane fluxes (CH_4_ flux), (**b**) pH, (**c**) total phosphorus concentrations (TP), (d) total nitrogen concentrations (TN), (**e**) diel temperature ranges (DTR), and (**f**) chlorophyll *a* concentrations (Chl *a*) from literature data extraction with optimal model fits from null, linear or null, segmented linear, and logistic curves in bold foreground lines. For nonlinear segmented and logistic models (**b**–**f**), plots are ordered by model breakpoints or inflection points (vertical background lines), indicative of boundaries between ponds and lakes.

**Figure 7:**
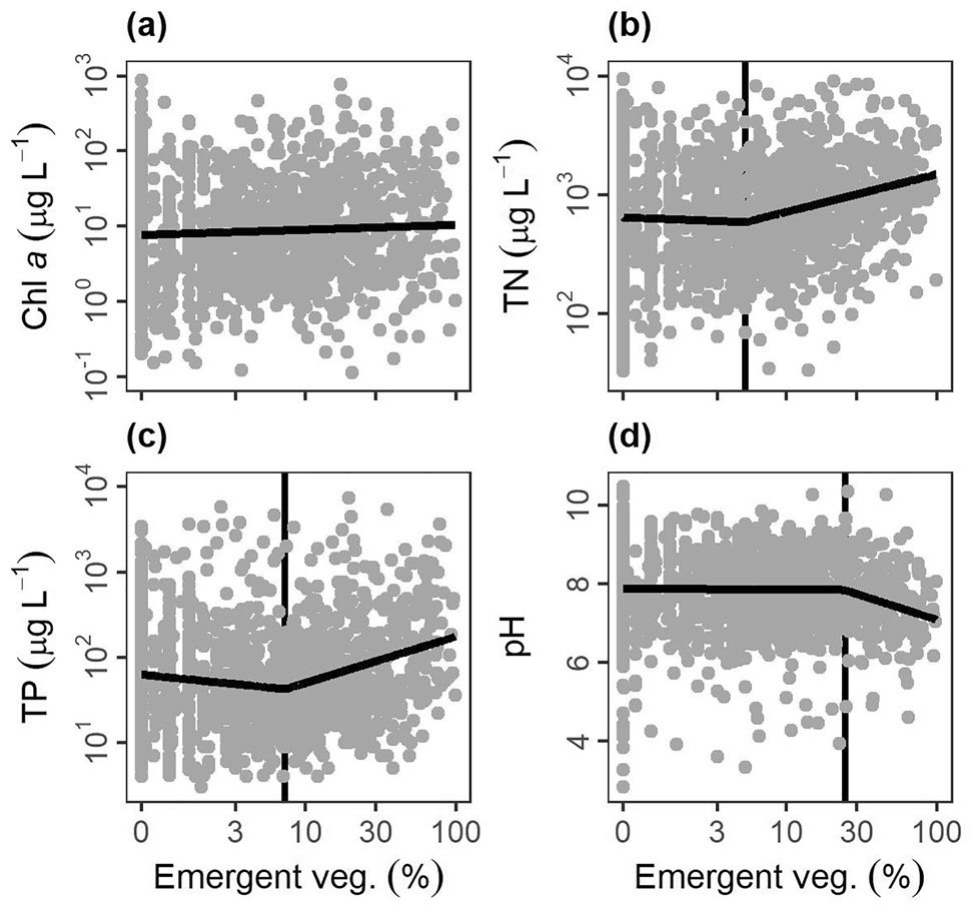
Relationships between lentic waterbody emergent vegetation cover (Emergent veg.) and various ecosystem structure and function metrics: (**a**) chlorophyll *a* concentrations (Chl *a*), (**b**) total nitrogen concentrations (TN), (**c**) total phosphorus concentrations (TP), (**d**) pH from literature data extraction with optimal model fits from null, linear or null, segmented linear, and logistic curves in bold foreground lines. For nonlinear segmented and logistic models (**b**–**d**), plots are ordered by model breakpoints or inflection points (vertical background lines), indicative of boundaries between ponds and wetlands.

Ecosystem structure and function were mostly nonlinearly related to surface area (n = 9/10 variables), depth (n = 5/6 variables), and emergent vegetation cover (n = 3/4 variables) with both segmented and logistic relationships occurring (Figs. 5, 6, 7; Tables S3–S8). For surface area, six variables had logistic relationships: TP (Fig. 5b), methane fluxes (Fig. 5d), chl *a* (Fig. 5f), diel temperature range (Fig. 5h), gas exchange rates (k_600_; Fig. 5i), and pH (not pictured). The inflection occurred at 0.8 ha for TP, 1.1 ha for methane fluxes, 1.5 ha for chl *a*, 1.7 ha for pH, 4.6 ha for diel temperature range, and 17.5 ha for gas exchange rates (Table S3). NEP (Fig. 5c), R (Fig. 5e), and TN (Fig. 5g) all had segmented linear relationships where smaller systems had steeper slopes than larger systems (Table S4). The breakpoint in surface area was 1.0 ha for NEP, 1.2 ha for R, and 3.8 ha for TN (Table S3). For depth, two variables had logistic relationships: diel temperature range (Fig. 6e) and chlorophyll a (Fig. 6f), with the inflection occurring at 5.9 m and 14.9 m, respectively (Table S5). pH (Fig. 6b), TP (Fig. 6c), and TN (Fig. 6d) all had segmented linear relationships where smaller systems had steeper slopes than larger systems (Table S6) with breakpoints occurring at 1.0, 2.1, and 5.2 m, respectively (Table S5). For emergent vegetation cover, TN (Fig. 7b), TP (Fig. 7c), and pH (Fig. 7d) all had segmented linear relationships where systems with more emergent vegetation had steeper slopes than more open systems (Table S8). The breakpoint in emergent vegetation cover was 6.0% for TN, 8.2% for TP, and 26.0% for pH (Table S7).

To summarize across all three metrics (surface area, depth, and emergent vegetation cover), we evaluated where the boundaries of nonlinear relationships generally occurred, which informs boundaries between ponds, lakes, and wetlands (Table 1). For surface area, the boundary was 3.7 ± 1.8 ha (mean ± standard error) and the median was 1.5 ha, consistent with the median of 2 ha from scientific definitions (Fig. 2b). The depth boundary was 5.8 ± 2.5 m (mean ± standard error) and the median was 5.2 m, within the range of scientific definitions (Fig. 2c). The emergent vegetation cover boundary was 13.4 ± 6.3% (mean ± standard error) and the median was 8.2%, both of which were lower than the previously identified wetland lower bound of 30%^31^.

**Table 1:** Nonlinear boundary values, parameter estimate ± standard error (SE), from comparisons between surface area, maximum (max.) depth, and emergent vegetation (veg.) cover and ecosystem structure/function metrics including gross primary production (GPP), total phosphorus concentrations (TP), methane fluxes (CH_4_ flux), respiration (R), net ecosystem production (NEP), chlorophyll *a* concentrations (Chl *a*), pH, total nitrogen concentrations (TN), diel temperature ranges (DTR), and gas transfer piston velocity (k_600_).

Ecosystem metric	Surface area Boundary est. ± SE (ha)	Max. depth Boundary est. ± SE (m)	Emergent veg. cover Boundary est. ± SE (%)
GPP	NA	–	–
TP	0.8 ± 1.2	2.1 ± 1.2	8.2 ± 1.2
NEP	1.0 ± 1.4	–	–
CH_4_ flux	1.1 ± 1.7	NA	–
R	1.2 ± 1.5	–	–
Chl *a*	1.5 ± 1.7	14.9 ± 1.2	NA
pH	1.7 ± 1.5	1.0 ± 1.4	26.0 ± 1.3
TN	3.8 ± 1.4	5.2 ± 1.4	6.0 ± 1.3
DTR	4.6 ± 1.3	5.9 ± 1.3	–
k_600_	17.5 ± 1.5	–	–
Mean	3.7 ± 1.8	5.8 ± 2.5	13.4 ± 6.3
Median	1.5	5.2	8.2

Boundary estimates are included if the nonlinear models (segmented regression or logistic relationships) were selected as optimal fits with standard error as determined when fitting the parameter. NA indicates a null or linear fit, – indicates not enough data was available to perform the analysis.

Pond morphology (e.g., size and depth) creates fundamentally distinct conditions that govern ecosystem structure and function. Specifically, ponds experience less wind-driven turbulence than larger waterbodies due to small fetch and sheltering from the landscape^36^. We found that gas exchange rates (k_600_) decreased at ~ 18 ha, presumably due to reduced wind shear (Fig. 5i; also supported by^37^) and altered thermal dynamics. For instance, ponds and shallow lakes can warm dramatically during the day, inducing stratification, and cool off and mix completely overnight^38^. We found higher diel temperature ranges were more common in waterbodies < 5 ha (Fig. 5h) and < 6 m (Fig. 6e; see also^39^). Such differences in temperature and mixing can promote internal nutrient loading^40^ and ecosystem respiration^41^, which may explain the higher TN (Figs. 4b, 5g), TP (Figs. 4a, 5b) and ecosystem respiration (Fig. 5e) found in ponds. Lastly, differences in water column mixing, increased nutrients, and higher respiration can all contribute to the higher greenhouse gas emissions found in ponds relative to lakes (Fig. 5d)^4,42^.

Metrics of phytoplankton biomass (chl *a*) and total ecosystem production in the water (GPP) exhibited weak or inconsistent relationships with surface area and depth, likely due to differences in the location and types of primary production across waterbody types. While total primary production in deep lakes is often dominated by phytoplankton^43^, shallow waterbodies can shift toward non-planktonic primary production like benthic algae or floating, emergent, or submerged macrophytes^44^. Ponds have pelagic phytoplankton, benthic algae (i.e., periphyton), and sediment rooted-submerged or floating macrophytes. In contrast, wetland productivity often predominantly occurs above the air–water interface^45^. Where emergent vegetation dominates, they may limit light and reduce water column nutrients, both of which are needed by phytoplankton and periphyton. Macrophytes can also modify water column and sediment geochemistry by providing autotrophic organic carbon and oxygen to rooting systems in the sediments^46^. Consequently, these opposing drivers can explain the high variability in primary production we observed (Fig. 5f, Table S2). Distinguishing ponds from wetlands will ultimately be aided by additional ecosystem measurements of metabolism, greenhouse gas production, and additional metrics (e.g., carbon burial) across shallow waterbodies with a range of emergent vegetation cover.

### A functional pond definition

Our review of existing pond definitions highlights that surface area and depth are the most common variables used to define ponds; yet how small and how shallow a waterbody must be to classify as a pond is unclear, with definitions ranging by orders of magnitude. Emergent vegetation is a third variable useful in distinguishing wetlands from ponds, but the threshold value, > 30% emergent vegetation coverage for wetlands established at the US federal level, is not based on documented changes in ecosystem function. Comparing characteristics among waterbodies that scientists self-categorized into lakes, ponds, or wetlands, ponds were sometimes distinct from lakes and wetlands (pH, TN), sometimes similar to wetlands (TP), and sometimes similar to lakes (chl *a*), suggesting ponds are an ecologically distinct type of ecosystem. Lastly, we found clear nonlinear relationships when we examined relationships between ecosystem structure or function and surface area, depth, and emergent vegetation cover; these boundaries help to quantitatively define ponds.

Specifically, we found that across available ecosystem metrics, ecosystems shift in structure and function at average (± SE) values of 3.7 (± 1.8) ha in size, 5.8 (± 2.5) m in depth, and 13.4 (± 6.3) % emergent vegetation cover (Table 1). For surface area, all but one ecosystem metric (*k*_600_) was below 5 ha in surface area, which fits well within the range of most existing definitions (≤ 10 ha; Fig. 2), and we suggest may be used to distinguish ponds from lakes. For maximum depth, all but one ecosystem metric (chl. *a*) was below a 6 m depth threshold, which also fits well within the range of depths reported in pond definitions (Fig. 2), and matches the published threshold of 5 m maximum depth for shallow lakes^44^. Our depth analysis was less robust than surface area because we had less depth data, a common challenge in lentic studies^47^; we therefore advise further studies in waterbodies to explicitly evaluate this threshold. Until further work is done, we recommend using 5 m as a maximum depth threshold for ponds as it is close to both threshold shifts in ecosystem function and matches with the shallow lake literature^44,48^. We had the fewest ecosystem metric comparisons for emergent vegetative cover, and observed three nonlinear boundaries ranging from 6 to 26% cover. The mean (13.4%), though smaller, is not statistically different than the 30% emergent vegetation cover (one sample t-test, t_2_ = − 2.6, p = 0.12) proposed by Cowardin et al.^31^ to separate wetlands from lakes. We recommend separating ponds and wetlands using the 30% coverage in emergent vegetation threshold for now, but recognize that the Cowardin et al.^31^ metric is not data driven and our analysis was limited by existing data. Future studies must examine how ecosystem structure and function shifts across a gradient of emergent vegetation cover to better functionally distinguish wetlands from ponds and could ultimately lower that boundary.

Our review of data from the literature showed scientists and managers view ponds as permanent or temporary and natural or human made in origin. Therefore, we felt it necessary to provide the inclusivity of these concepts in a pond definition. Other definitions also link depth to light availability, where light penetrates to the sediments across the pond (e.g.,^11^). However, light availability is not only mediated by depth; even in the shallowest systems light can be limiting due to turbidity, dissolved organic matter, and submerged or floating plants (e.g.,^49,50^). For example, floating duckweed can cover most of a pond’s surface area and reduce light penetration to < 1% relative to the light above the water’s surface^49^, and dramatically change the ecology of shallow systems^51^.

**Figure 8:**
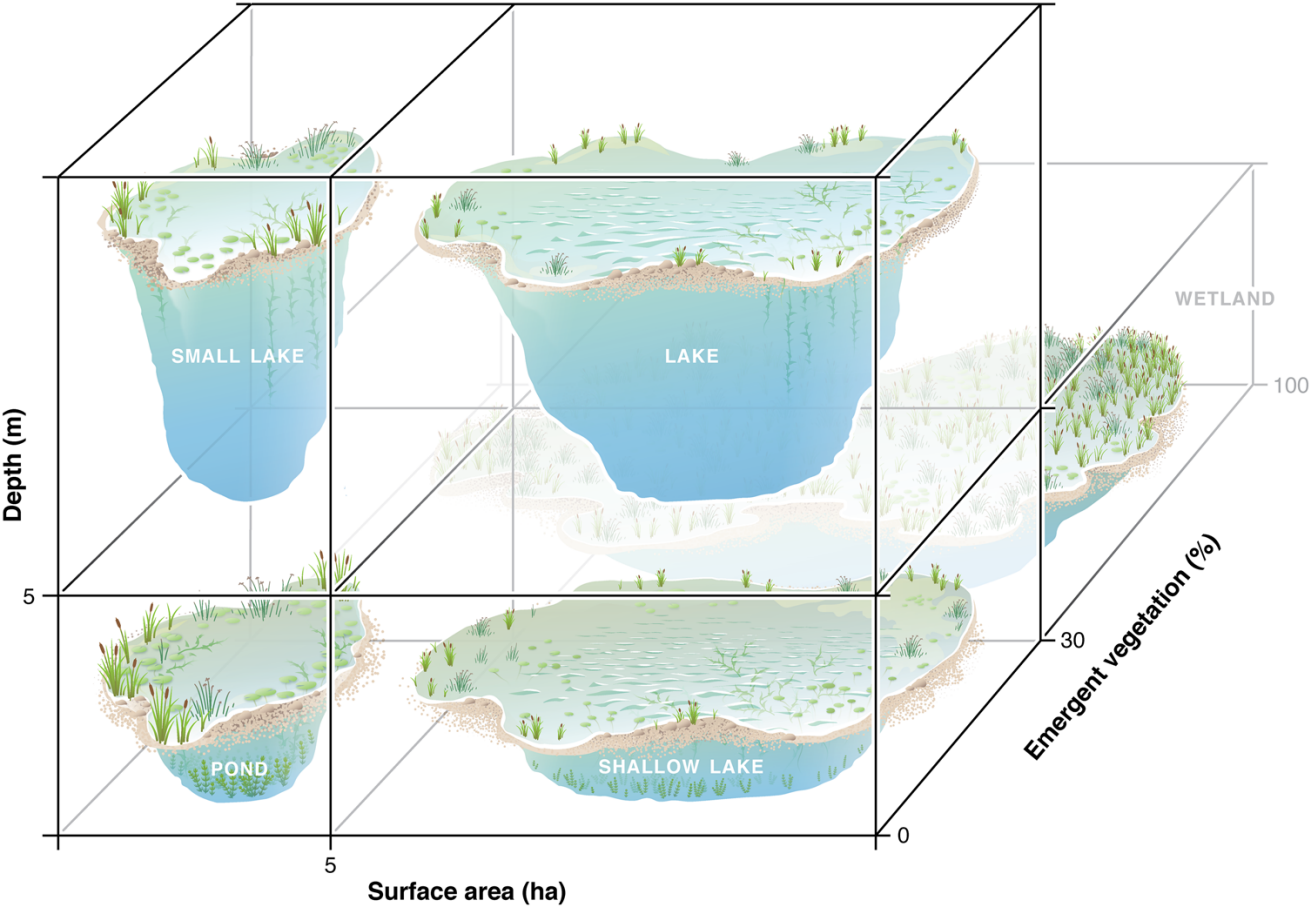
Conceptual model to define lentic waterbodies based on three different criteria (depth, surface area, and emergent vegetation). Boundaries for all three axes come from our analysis and are informed by existing pond, lake, and wetland definitions. Figure by Visualizing Science.

As our analyses indicate that ponds are functionally distinct from lakes and wetlands, we propose the following scientifically informed pond definition (Fig. 8):

Ponds are small and shallow waterbodies with a maximum surface area of 5 ha, a maximum depth of 5 m, and < 30% coverage of emergent vegetation. Ponds will have light penetration to the sediments if water clarity permits and can be permanent or temporary and natural or human-made.

Our proposed definition is based on the current state of the science; we anticipate that future research will further resolve differences among these five categories. For example, we call for future research to examine how ecosystem structure and function shift across our proposed boundaries, particularly for depth and emergent vegetation, which had smaller sample sizes and fewer ecosystem metrics than surface area. Additional variables such as basin geometry (e.g., area:volume ratios), sheltering from wind, water residence time, water clarity, and geographic location, may also affect a waterbody’s ecosystem structure and function, creating some overlap between classifications especially along the upper and lower bounds of our pond definition. For instance, a landscape with little wind sheltering increases water column mixing that could cause a waterbody the size of a pond to function more like a shallow lake. We therefore advocate for additional sampling of lentic waterbodies, especially in locations where lentic waterbodies are understudied or being rapidly constructed like tropical and subtropical regions^52^, to help resolve boundaries among waterbody types and further refine the pond definition.

## Conclusion

Scientists, policy makers, water resource managers and the public all use the word “pond” to describe small and shallow waterbodies, which are globally abundant^8^ and hotspots for biogeochemistry^4,8^ and biodiversity^53^. Yet, the lack of a universal pond definition means that ponds can fall between lake and wetland jurisdictions and categorizations^22^, thus potentially limiting their legal protections. Globally, the situation is similar to US policy as some nations define ponds as wetlands (e.g., the Ramsar Convention), some as lakes (e.g., Denmark), and others specifically define ponds (e.g., United Kingdom). The pond definition presented here will favor more frequent and consistent use of the term and ultimately improve the protection, monitoring, and scientific study of ponds, which are globally abundant and structurally and functionally distinct from other lentic waterbodies.

## Methods

### Literature survey

To compile biological, physical, and chemical characteristics of ponds, we conducted a literature search based on three seminal papers establishing the ecological importance of ponds: Oertli et al.^35^, Søndergaard et al.^18^, and Downing^8^, each of which has > 100 citations and is more than ten years old. We conducted a backwards and forwards search in April 2019 to compile all papers cited by these three papers, and all papers that cited them, yielding 519 unique papers. We extracted physical, chemical, and biological data for papers that reported data for individual waterbodies defined as ponds by the publishing scientists. To ensure consideration of all potential ponds, we checked that waterbodies selected were small (≤ 20 ha in surface area) and shallow (≤ 9 m in maximum or mean depth), boundaries that are slightly greater than the maximum depth (8 m)^35^ and maximum surface area (10 ha)^34^ used to define ponds in a few prior studies. We used the resulting 1327 waterbodies in our analysis, which had a global distribution (Fig. S1)^19^.

### Scientific definitions

To investigate how scientific researchers defined ponds, we reviewed all 519 papers for pond definitions. We included definitions where the authors explicitly referred to their study waterbodies as ponds (e.g., we excluded “shallow lakes” and “small lakes”), yielding 40 pond definitions. The definitions included 89 citations of 48 unique papers; we evaluated all cited papers that were not already in our compilation for additional definitions and citations. This process added 14 definitions, plus an additional five cited papers not assessed due to our inability to access or translate them (data available^19^).

### Federal and state definitions

We examined policy definitions using the United States (US) federal and state legislation as an example because we posited differences at this scale would reflect the challenges faced by governments from other countries in formulating a unified pond definition. At the federal level, we examined three agencies with monitoring or regulatory responsibilities: US Environmental Protection Agency (EPA), US Army Corps of Engineers (ACE), and US Fish and Wildlife Service (USFWS). Due to the difficulty of finding all state policies, we sent electronic surveys to individuals working in state environmental agencies in all states. We asked whether their state defined lakes, ponds, and wetlands, and requested the legislative sources. We received responses from 42/50 states and evaluated all definitions provided and their associated legislation.

### Pond, lake, and wetland data

We compared chemical and biological characteristics among various lentic waterbodies as defined by scientists as ponds, wetlands, or lakes. For ponds, we used data from the literature data extraction as described above (n = 1327). Wetland data came from the US EPA’s 2011 National Wetland Condition Assessment, which surveyed wetlands with standing water < 1 m in depth and variable surface area^54,55^. We selected wetland sites that were freshwater and had water chemistry data (n = 400). Lake data was extracted from LAGOS-NE (lakes ≥ 4 ha; n = 51,101)^56^, EPA’s 2012 National Lake Assessment (lakes ≥ 1 ha; n = 1130)^57,58^, and the European Environmental Agency’s Waterbase database (lakes > 0.02 ha; n = 2942)^59^. From these sources, we compared nutrients (total phosphorus (TP), total nitrogen (TN)), water chemistry (pH), and primary producer biomass (chl *a*) among waterbody types (ponds, wetlands, and lakes).

We also examined differences in six additional metrics of ecosystem function across waterbodies using data from a variety of sources and ranging in sample size from 67 to 198 global sites (gross primary production—GPP, respiration—R, net ecosystem production—NEP, diel temperature ranges—DTR, methane fluxes, gas transfer velocities). We extracted metabolism metrics (GPP and R) from an existing literature review^60^ and two published studies of various sized lentic ecosystems^41,61^. DTR, calculated as the diel difference between the maximum and minimum surface temperature for each waterbody, were extracted from multiple studies^38,39^. Areal methane fluxes^42^ and gas transfer velocities (k_600_)^37^ were extracted from existing literature reviews.

### Comparing lake, pond, and wetlands characteristics from literature

We evaluated whether there were differences among waterbody types as defined by scientists and managers. We determined significant differences in waterbody characteristics across waterbody types using ANOVA and post-hoc Least Significant Difference (LSD) analysis. We determined the variation within each freshwater type using the coefficient of variation (cv) and tested for significant differences using Levene’s test. We acknowledge that the confounding definitions of waterbodies resulted in overlapping size distributions; therefore any statistical differences across waterbody types will be conservative.

### Does pond ecosystem structure and function distinguish ponds differ from lakes and wetlands?

We evaluated where the cutoffs might exist along pond to lake and pond to wetland gradients. We used the relationship between surface area, depth, or emergent vegetation cover represented by *x* below and each ecosystem variable (n = 10 variables for surface area; n = 6 variables for depth, n = 4 for emergent vegetation cover) represented by *y* below for four different patterns in increasing order of complexity: null, linear, segmented (nonlinear), and logistic (nonlinear) patterns. We fit null models by taking the arithmetic mean (Eq. 1), linear models using ordinary least-squares linear regression (Eq. 2), segmented using regressions with one breakpoint (Eq. 3) via the “segmented” package^62^, and logistic using sigmoidal curves (Eq. 4) via the *nls* function in R (Fig. S2). Parameters *a* – *h* and *bp* (breakpoint) were fit using the methods above.

1 $$y=a$$

2 $$y=bx+c$$

3 $$y=\{{d}_{1}*x+{e}_{1}, x\le bp\ {d}_{2}*x+{e}_{2},x>bp$$

4 $$y=f+\frac{g-f}{1+{e}^{(bp-x)/h}}$$

We log-transformed surface area and depth to account for the several order of magnitude scale and non-normality. Similarly, we transformed some of the ecosystem variables depending on normality and distributions. To select among the four models for each relationship, we examined the AICc fits and selected the minimum AICc as the optimal fit with consideration of other model fits within 11 units of the minimum AICc using root mean squared error and parsimony^63^. If one of the nonlinear models was selected, we then objectively quantified the boundary among ecosystem types (i.e., pond vs. lake or pond vs. wetland) using either the breakpoint or the inflection point parameter from the segmented regression or sigmoid curve, respectively (Fig. S2c,d).

## Supplementary Information

Supplementary Information.

## Data Availability

All data used for this manuscript is available through an Environmental Data Initiative data publication (Richardson et al. 2022: https://doi.org/10.6073/pasta/ec507ac70846b17d0633d95aa3c680c6).
