# Supplementary material for: A functional definition to distinguish ponds from lakes and wetlands
Source: Sci Rep. 2022 Jun 21;12:10472. doi: 10.1038/s41598-022-14569-0 (PMC9213426; doi:10.1038/s41598-022-14569-0)
Supplement: Supplementary file 1 — Supplementary Information. [file 41598_2022_14569_MOESM1_ESM.pdf]

Title: A functional definition to distinguish ponds from lakes and wetlands

Authors:

David C. Richardson[=*], Meredith A. Holgerson[=], Matthew J. Farragher, Kathryn K. Hoffman, Katelyn B.S. King, María B. Alfonso, Mikkel R. Andersen, Kendra Spence Cheruveil, Kristen A. Coleman, Mary Jade Farruggia, Rocio Luz Fernandez, Kelly L. Hondula, Gregorio A. López Moreira Mazacotte, Katherine Paul, Benjamin L. Peierls, Joseph S. Rabaey, Steven Sadro, María Laura Sánchez, Robyn L. Smyth, Jon N. Sweetman

[=] Co-first authors contributed equally to the conception, design, analysis, and writing of this paper

[*] Indicates corresponding author, richardsond@newpaltz.edu

Supplementary Information

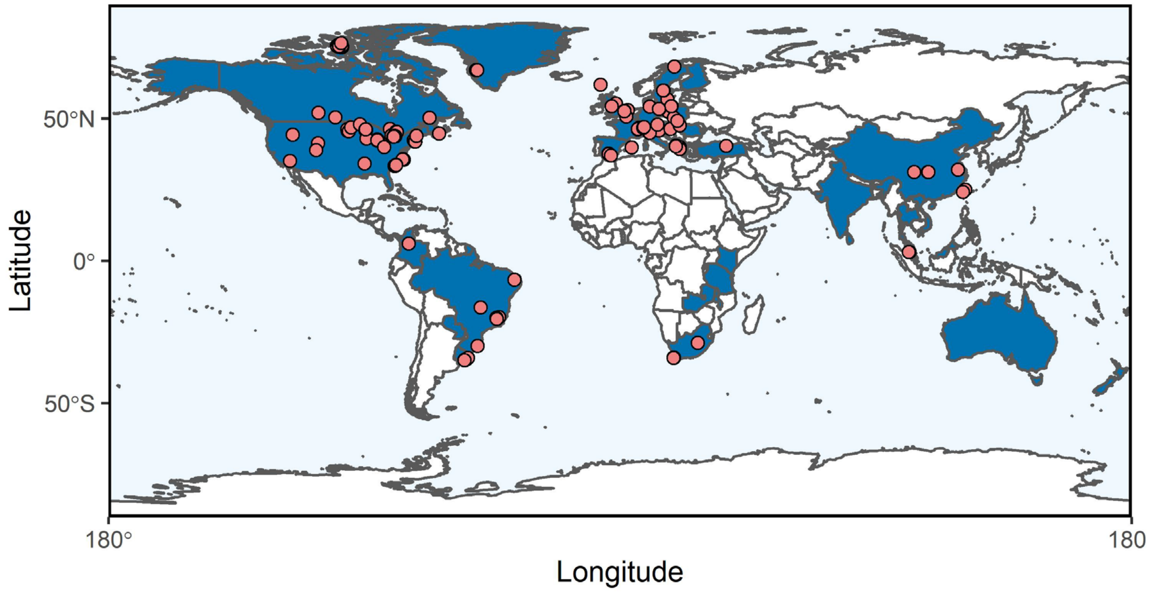

Figure S1. Global locations of waterbodies from the literature search that were used in analysis. Note that only those sites where latitude and longitude were included could be mapped (n = 591 of 1327) as red circles. If country of origin was available for a pond but individual latitude and longitude were not, the country is indicated in blue. The figure was generated in R (R Core Team 2020) using packages ggplot2 (Wickham 2016) and sf (Pebesma 2018).

H. Wickham. 2016. ggplot2: Elegant Graphics for Data Analysis. Springer-Verlag New York.

Pebesma, E., 2018. Simple Features for R: Standardized Support for Spatial Vector Data. The R Journal 10 (1), 439-446, https://doi.org/10.32614/RJ-2018-009

R Core Team. 2020. R: A language and environment for statistical computing. R Foundation for Statistical Computing, Vienna, Austria. URL https://www.R-project.org/.

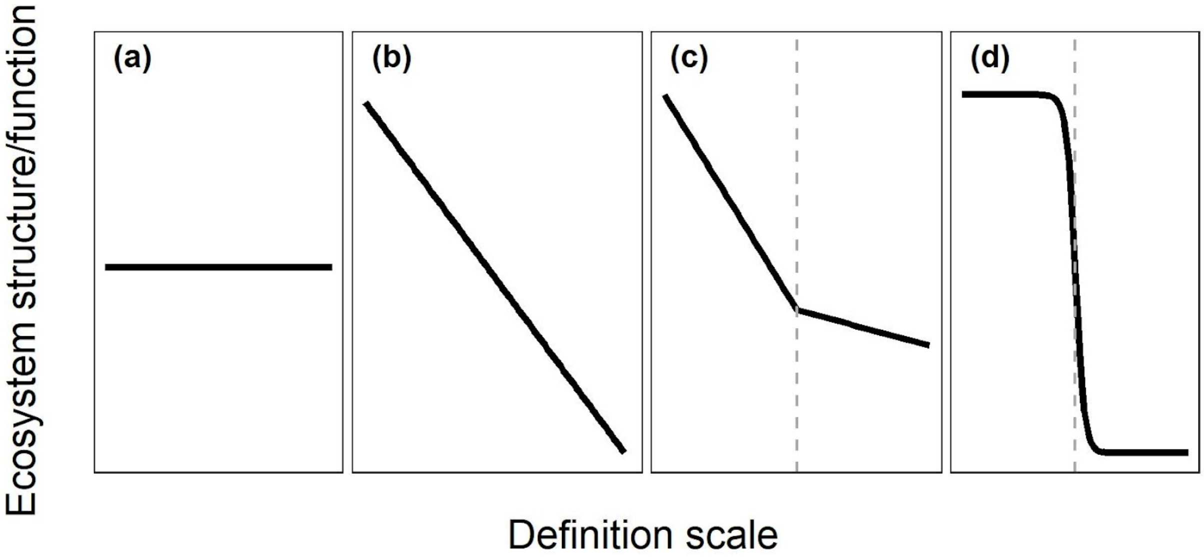

Figure S2. Theoretical curves for possible linear and nonlinear relationships between defining variables (definition scale, e.g., surface area, depth, vegetative cover) and ecosystem structure or function. Relationships could be (a) non-existent or null (Eq. 1), (b) linear (Eq. 2), or nonlinear with different curve shapes: (c) segmented linear (Eq. 3), or (d) logistic (Eq. 4). For nonlinear models, boundaries (dotted grey line) are defined statistically by breakpoints or inflection points as defined by *bp* in the equations below with *x* representing the definition scale, *y* representing the ecosystem structure or function metrics, and letters *a* through *h* representing parameters fit by statistical modeling explaining in the main text.

Table S1: Statistics of comparisons among lakes, ponds, and wetlands. ANOVA results for significant difference in means across waterbody types and Levene's test results for significance in variation within waterbody type.

Response variable	DF	ANOVA		Levene's test	
		F	*p*	F	*p*
TP (µg/l)	2, 10346	733.7	**<0.001**	350.1	**<0.001**
TN (µg/l)	2, 5649	147.2	**<0.001**	11.0	**<0.001**
pH	2, 4690	176.5	**<0.001**	20.7	**<0.001**
Chl *a* (µg/l)	2, 10983	66.5	**<0.001**	299.0	**<0.001**

Table S2: Statistics of comparisons among lakes, ponds, and wetlands. Coefficient of variation within waterbody type and means of each variable across waterbody types. Letters for significant differences between types from LSD are in Figure 4.

Response variable	n			Mean			Coefficient of variation		
	lake	pond	wetland	lake	pond	wetland	lake	pond	wetland
TP (μg/l)	9476	473	400	24	129	134	372	502	221
TN (μg/l)	5025	237	390	605	1293	1081	162	163	228
pH	3790	559	347	7.85	7.40	7.02	11	14	14
Chl *a* (μg/l)	10166	427	393	99	82	36	180	277	384

Table S3. Statistics from multiple comparisons between surface area and ecosystem structure/function metrics including gross primary production (GPP), total phosphorus concentrations (TP), methane fluxes (CH_4 flux), respiration (R), net ecosystem production (NEP), chlorophyll *a* concentrations (Chl *a*), pH, total nitrogen concentrations (TN), diel temperature ranges (DTR), and gas transfer piston velocity (k_{600}). Log trans. column indicates if the ecosystem metric was log10-transformed (Yes/No). Akaike information criterion with correction for model fits are presented with the AICc in bold for the selected model and in italics for the models within 11 units of the minimum AICc. A surface area boundary (ha) is included if the nonlinear models were selected as optimal fits with standard error as determined when fitting the parameter.

Ecosystem metric	Log trans.	N	AICc null	AICc linear	AICc segmented	AICc logistic	Boundary est. ± SE (ha)
GPP[1]	N	100	***1207.1***	*1206.4*	*1207.1*	1221.6	NA
TP	Y	7801	12775.2	12489.6	12186.5	***12131.0***	0.8±1.2
NEP	N	100	1083.2	*1062.2*	***1053.6***	1066.6	1.0±1.4
CH_4 flux[2]	Y	198	470.7	441.1	*428.1*	***428.8***	1.1±1.7
R	N	100	1239.0	*1228.1*	***1221.5***	*1230.0*	1.2±1.5
Chl *a*	Y	8597	14653.0	14641.3	*14617.3*	***14614.3***	1.5±1.7
pH	N[3]	1574	4470.9	4263.8	4243.8	***4231.6***	1.7±1.5
TN	Y	3854	3550.7	3378.3	***3309.6***	*3312.5*	3.8±1.4
DTR	N	143	636.3	563.6	562.0	***542.1***	4.6±1.3
k_{600}	Y	67	42.0	*21.5*	22.7	***11.4***	17.5±1.5

[1]The null model was within 2 AICc units of the linear model and we chose the most parsimonious model.
[2]The logistic model was within 0.7 units of the segmented model. Residual standard error for both models was 0.7. We choose the logistic model which put less emphasis on the larger waterbodies. For the segmented model, the boundary was 116.4 ha. If 116.4 ha was used as the boundary for CH_4 flux, the aggregate surface area boundary would be 16.5 ± 12.6 ha (mean ± standard error) and the median would be 1.74 ha.
[3]pH units are already log transformed.

Table S4. Optimal relationships between surface area (SA) and each ecosystem structure/function metric including gross primary production (GPP), total phosphorus concentrations (TP), net ecosystem production (NEP), methane fluxes (CH_4 flux), respiration (R), chlorophyll *a* concentrations (Chl *a*), total nitrogen concentrations (TN), diel temperature ranges (DTR), and gas transfer piston velocity (k_{600}).

Ecosystem metric	Units	Optimal fit	Equation
GPP	mmol m^{-3} day^{-1}	Null	87.1
TP	μg P L^{-1}	Logistic	$1.4 + \frac{2.5 - 1.4}{1 + e^{(-0.1 - \log(SA))/-0.3}}$
NEP	mmol m^{-3} day^{-1}	Segmented	$\begin{cases} -28 + 154 * \log(SA), SA \le 1.0 \\ -28 + 12.0 * \log(SA), SA > 1.0 \end{cases}$
CH_4 flux	m d^{-1}	Logistic	$1.1 + \frac{2.1 - 1.1}{1 + e^{(0.03 - \log(SA))/-0.4}}$
R	mmol m^{-3} day^{-1}	Segmented	$\begin{cases} 133 - 272 * \log(SA), SA \le 1.2 \\ 113 - 16.0 * \log(SA), SA > 1.2 \end{cases}$
Chl *a*	μg L^{-1}	Logistic	$0.9 + \frac{1.2 - 0.9}{1 + e^{(0.2 - \log(SA))/-0.3}}$
pH	pH units	Logistic	$8.2 + \frac{7.0 - 8.2}{1 + e^{(0.2 - \log(SA))/-0.6}}$
TN	μg N L^{-1}	Segmented	$\begin{cases} 2.95 - 0.25 * \log(SA), SA \le 3.8 \\ 2.83 - 0.04 * \log(SA), SA > 3.8 \end{cases}$
DTR	°C	Logistic	$0.9 + \frac{4.7 - 0.9}{1 + e^{(0.7 - \log(SA))/-0.2}}$
k_{600}	mg C m^{-2} d^{-1}	Logistic	$0.1 + \frac{-0.4 - 0.1}{1 + e^{(1.2 - \log(SA))/-0.3}}$

Table S5. Statistics from multiple comparisons between maximum depth and ecosystem structure/function metrics including methane fluxes (CH_4 flux), pH, total phosphorus concentrations (TP), total nitrogen concentrations (TN), diel temperature ranges (DTR), chlorophyll *a* concentrations (Chl *a*). Other variables like metabolism and gas transfer piston velocity variables used for relationships with size did not have sufficient depth data. Log trans. column indicates if the ecosystem metric was log10-transformed (Yes/No). Akaike information criterion with correction for model fits are presented with the AICc in bold for the selected model and in italics for the models within 11 units of the minimum AICc. A depth boundary (m) is included if the nonlinear models were selected as optimal fits with standard error as determined when fitting the parameter.

Ecosystem metric	Log trans.	N	AICc null	AICc linear	AICc segmented	AICc logistic	Boundary est. ± SE (m)
CH_4 flux[1]	Y	135	315.6	***293.5***	*291.4*	*295.5*	NA
pH	N[2]	1329	3564.5	3565.4	***3546.8***	3569.9	1.0±1.4
TP	Y	7199	10297.8	8691.2	***8670.5***	8729.4	2.1±1.2
TN	Y	3395	3021.4	*2211.7*	***2208.4***	*2213.4*	5.2±1.4
DTR	N	137	612.5	*526.1*	*523.6*	***520.6***	5.9±1.3
Chl *a*	Y	7931	13387.6	12342.7	*12269.4*	***12269.2***	14.9±1.2

[1]The null model was within 2 AICc units of the linear model and we chose the most parsimonious model.
[2]pH units are already log transformed.

Table S6. Optimal relationships between depth (D) and each ecosystem structure/function metric including methane fluxes (CH_4 flux), pH, total phosphorus concentrations (TP), total nitrogen concentrations (TN), diel temperature ranges (DTR), chlorophyll *a* concentrations (Chl *a*).

Ecosystem metric	Units	Optimal fit	Equation
CH_4 flux	m d^{-1}	Linear	$1.7 - 0.6 * D$
pH	pH units	Segmented	$\begin{cases} 8.1 + 1.1 * \log(D), D \leq 1.0 \\ 8.1 - 0.1 * \log(D), D > 1.0 \end{cases}$
TP	µg P L^{-1}	Segmented	$\begin{cases} 1.9 - 0.9 * \log(D), D \leq 2.1 \\ 1.8 - 0.5 * \log(D), D > 2.1 \end{cases}$
TN	µg N L^{-1}	Segmented	$\begin{cases} 3.1 - 0.5 * \log(D), D \leq 5.2 \\ 3.0 - 0.4 * \log(D), D > 5.2 \end{cases}$
DTR	°C	Logistic	$0.7 + \frac{5.0 - 0.7}{1 + e^{(0.8 - \log(D))/-0.3}}$
Chl *a*	µg L^{-1}	Logistic	$0.2 + \frac{1.3 - 0.2}{1 + e^{(1.2 - \log(D))/-0.4}}$

Table S7. Statistics from multiple comparisons between emergent vegetation cover and ecosystem structure/function metrics including methane fluxes (CH_4 flux), pH, total phosphorus concentrations (TP), total nitrogen concentrations (TN), diel temperature ranges (DTR), chlorophyll *a* concentrations (Chl *a*). Other variables like metabolism and gas transfer piston velocity variables used for relationships with size did not have sufficient depth data. Log trans. column indicates if the ecosystem metric was log10-transformed (Yes/No). Akaike information criterion with correction for model fits are presented with the AICc in bold for the selected model and in italics for the models within 11 units of the minimum AICc. A surface area boundary (ha) is included if the nonlinear models were selected as optimal fits with standard error as determined when fitting the parameter.

Ecosystem metric	Log trans.	N	AICc null	AICc linear	AICc segmented	AICc logistic	Boundary est. ± SE (m)
Chl *a*	Y	1384	*2916.6*	***2914.1***	*2914.1*[1]	2950.5	NA
TN	Y	1506	2015.6	1976.4	***1958.6***	*1959.3*	6.0±1.3
TP	Y	1516	2813.0	2799.4	***2752.9***	2765.4	8.2±1.2
pH	Y[2]	1462	4145.5	4136.6	***4124.8***	*4125.3*	26.0±1.3

[1]No breakpoint was selected for the model.

[2]pH units are already log transformed.

Table S8. Optimal relationships between percent emergent vegetation cover (E) and each ecosystem structure/function metric including methane fluxes (CH_4 flux), pH, total phosphorus concentrations (TP), total nitrogen concentrations (TN), diel temperature ranges (DTR), chlorophyll *a* concentrations (Chl *a*).

Ecosystem metric	Units	Optimal fit	Equation
Chl *a*	µg L^{-1}	Linear	$0.88 - 0.07 * \log(E)$
TN	µg N L^{-1}	Segmented	$\begin{cases} 2.8 - 0.05 * \log(E), E \leq 6.0 \\ 2.5 + 0.38 * \log(E), E > 6.0 \end{cases}$
TP	µg P L^{-1}	Segmented	$\begin{cases} 1.8 - 0.19 * \log(E), E \leq 8.2 \\ 0.9 + 0.75 * \log(E), E > 8.2 \end{cases}$
pH	pH units	Segmented	$\begin{cases} 7.9 - 0.02 * \log(E), E \leq 26 \\ 9.6 - 1.28 * \log(E), E > 26 \end{cases}$
